# Advanced Bionic Attachment Equipment Inspired by the Attachment Performance of Aquatic Organisms: A Review

**DOI:** 10.3390/biomimetics8010085

**Published:** 2023-02-16

**Authors:** Dexue Zhang, Jin Xu, Xuefeng Liu, Qifeng Zhang, Qian Cong, Tingkun Chen, Chaozong Liu

**Affiliations:** 1 Key Laboratory of Bionic Engineering, Ministry of Education, Jilin University, Changchun 130022, China; 2 Shandong Academy of Agricultural Machinery Sciences, Jinan 250100, China; 3 Institute of Modern Agriculture on Yellow River Delta, Shandong Academy of Agricultural Sciences, Dongying 257300, China; 4 State Key Laboratory of Automotive Simulation and Control, Jilin University, Changchun 130022, China; 5 Institute of Orthopaedic & Musculoskeletal Science, University College London, London HA7 4LP, UK

**Keywords:** aquatic organisms, classification, attachment mechanisms, biomimetic equipment, potential applications

## Abstract

In nature, aquatic organisms have evolved various attachment systems, and their attachment ability has become a specific and mysterious survival skill for them. Therefore, it is significant to study and use their unique attachment surfaces and outstanding attachment characteristics for reference and develop new attachment equipment with excellent performance. Based on this, in this review, the unique non-smooth surface morphologies of their suction cups are classified and the key roles of these special surface morphologies in the attachment process are introduced in detail. The recent research on the attachment capacity of aquatic suction cups and other related attachment studies are described. Emphatically, the research progress of advanced bionic attachment equipment and technology in recent years, including attachment robots, flexible grasping manipulators, suction cup accessories, micro-suction cup patches, etc., is summarized. Finally, the existing problems and challenges in the field of biomimetic attachment are analyzed, and the focus and direction of biomimetic attachment research in the future are pointed out.

## 1. Introduction

In recent years, vacuum attachment technology has been widely used in many important fields such as industry, aviation, and medicine due to its stability, practicality, and cleanliness advantages [1,2,3]. Based on the negative pressure vacuum technology, various types of vacuum attachment equipment such as suction cup robots, suction cup assemblies, and suction cup patches have emerged as required and have brought a lot of convenience to people’s production and life. For example, suction cup robots can help humans complete multiple tasks such as transportation, packaging, and monitoring. Vacuum sucker assemblies can be integrated with robots, manipulators, etc., and play an important role in many fields such as product packaging, logistics, and transportation. In addition, various micro-suction cup patches developed in recent years have been widely used in the frontier fields of biology, electronics, medicine, etc. because of their good biocompatibility and environmental friendliness. However, these vacuum attachment devices are not perfect at present, and they still have their shortcomings. For example, the technologies of flexible sensing, driving, and control adopted by sucker robots are not mature enough. When working in complex underwater or harsh environments, they are very likely to become out of control [4]. Similarly, when vacuum suction cup assemblies and micro-suction cup patches are used for attachment in a wet or complex environment, problems such as lateral sliding and poor sealing may also lead to short attachment time and poor attachment effect [5]. Therefore, it is urgent to explore the failure causes of attachment equipment on non-smooth surfaces in complex environments and to develop more advanced attachment equipment with wider adaptability, better attachment performance, higher reliability, etc.

Biomimicry is a newly emerging interdisciplinary subject arising from the rapid development of biology and technical sciences. Its purpose is to learn from nature and transform the world [6,7]. Therefore, people can adopt the idea of bionics to solve the attachment problems of the current attachment equipment through in-depth exploration and reference to the unique attachment characteristics of natural organisms. The attachment behavior of organisms is a ubiquitous adaptive evolutionary movement pattern, which exists widely in nature and has been mastered and used by many organisms [8]. For hundreds of millions of years, organisms have gradually evolved biological suction cups with different shapes and functions to compete in nature. Surprisingly, even living in a complex underwater environment, aquatic attachment organisms can also use their suction cups to complete various attachment tasks such as movement, foraging, and finding a mate excellently, as animals do on land [9,10]. For example, the octopus in the deep sea can complete many jobs, including movement, hunting, environment perceiving, and escaping in distress, owing to the high-performance attachment capacity of thousands of small suction cups distributed on its arms [11,12,13]. The remora can attach to the bottom of a ship or the surfaces of turtles, sharks, and other marine life, and hitch a ride around the ocean with the help of its suction cup on the head (back) [14]. *Sinogastromyzon szechuanensis* can be firmly attached to irregular rough rock surfaces in turbulent water with a suction system composed of lips, pectoral fins, pelvic fins, and needle-like setae [15]. In addition, the suckermouth catfish and *Gyrinocheilus aymonieri* living in freshwater can attach to and feed on tank walls and stones with the closed sucker-like structures of their mouths [16,17]. In a word, aquatic attachment organisms have unique attachment morphologies and excellent attachment capacity. Hence, their suction cup structures and attachment mechanisms are worth exploring, and their extraordinary attachment ability is also worth our reference.

At present, the research on the unique structures and mechanisms of underwater biological suction cups is still under continuous exploration, and the corresponding bionic attachment equipment has also achieved specific results. For instance, various artificially integrated suction arms with sticky suction cups, free-swimming biorobots, and multi-functional skin patches have been developed by imitating the octopus’s suction cups [18,19,20]. The first and second generations of bionic attachment robots have been developed through the exploration of the attachment mechanism of the remora. The first-generation bionic attachment robot can attach to moving objects to hitchhike underwater while the second generation, which is better, can realize cross-border flight and attachment fixation both underwater and in the air [21,22]. The loadable bidirectional crawling bionic robot that is inspired by rock-climbing fish can achieve free forward and backward movement in the horizontal, vertical, and inverted planes [23]. However, as mentioned above, there are also some shortcomings to be solved in the current attachment equipment. For example, the attachment equipment in deep sea, closed, narrow, and other complex environments still struggles to complete long-term attachment work. Therefore, it is necessary to accelerate the pace of exploration and scientific research, pay attention to references, forge ahead with innovation, and develop more high-quality bionic attachment equipment, which will benefit people worldwide.

In this review, the surface morphologies of the suckers are classified firstly based on the unique non-smooth surface structures and morphologies of aquatic attachment biological suckers. Combined with the related research on the surface morphologies and the test experiments on the attachment capacity of marine organisms in recent years, their mechanisms and internal laws of attachment are introduced. Then, the newly developed research progress of advanced bionic attachment in recent years is summarized. Finally, the existing problems and challenges are analyzed, and the application prospect of bionic attachment research in the future is pointed out.

## 2. Non-Smooth Structural Morphologies and Attachment Mechanisms of Aquatic Organisms

After millions of years of continuous evolution, aquatic attachment organisms have evolved various structures and morphologies of non-smooth attachment surfaces, mainly including small suckers, hairs, sticky pads, grooves, dimples, papillae, etc. Although the attachment structures and mechanisms of aquatic organisms have different characteristics, they have commonality and regularity for the common purpose of their attachment stability. It is important to note that multi-level structures of cross-level size have been found in the attachment structures of some aquatic organisms by magnifying them with a microscope. In addition, most of these multi-level structures appear on hair structures, so they are also called multi-level hair-like structures. Studies have shown that these multi-level hair-like structures are critical and can significantly enhance the underwater attachment capacity of aquatic organisms, which should be worthy of our attention [15]. Therefore, through analysis and summary, various non-smooth attachment morphological characteristics of aquatic organisms can be divided into single-level non-smooth structures and multi-level hair-like structures according to whether the sucker structures of the aquatic organisms have these unique structures. In addition, their important roles and mechanism in the process of biological attachment can also be discussed and explored.

### 2.1. The Single-Level Non-Smooth Structures of Aquatic Organisms and Their Attachment Mechanisms

The single-level non-smooth structures refer to a single surface structure of a microscopic structure after magnification by a microscope. They mainly include single-level structures with gullies, spinous (barb)-like structures, and many others.

#### 2.1.1. The Gully-Shaped Single-Level Structures

Many aquatic attachment biosorption surfaces have single-level structures with gullies. The gully-shaped single-level structures mainly include folds, pits, grooves, etc. Typical aquatic attachment organisms with gully-like structures include the octopus, the leech, the abalone, the hill loach, etc.

**Figure 1:**
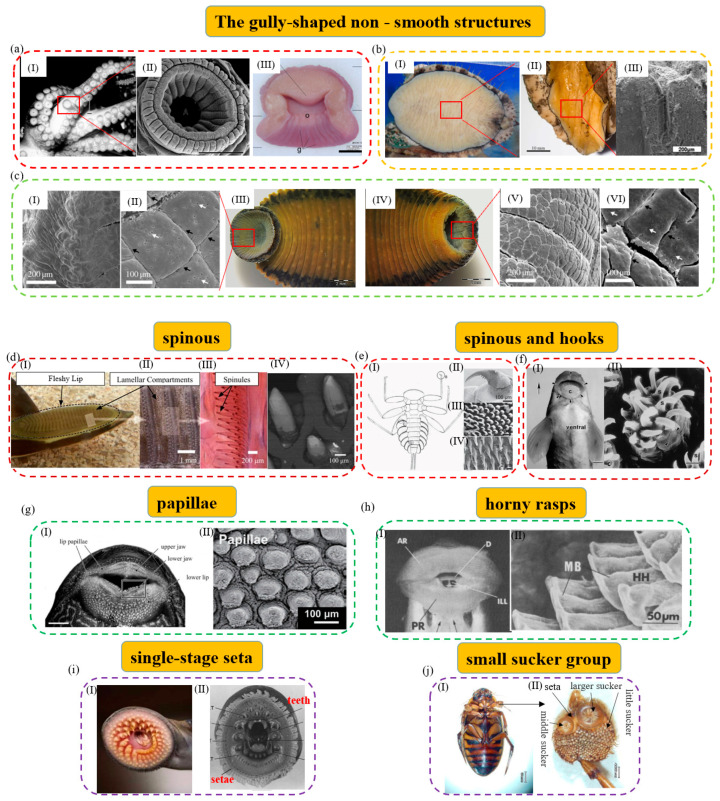
The single−level non−smooth surface structures of typical aquatic organisms. (**a**) The octopus and its fold structure of funnel body (reproduced with permission from [11,32], IOP Publishing Ltd., Copyright 2015, and Oxford University Press, Copyright 2002, respectively): (I) The octopus is attached to the aquarium wall, showing the general form and arrangement of its suckers; (II) SEM image of the octopus’s sucker. There are lots of radial grooves and ridges on its infundibulum; (III) transverse section of sucker of the octopus. (**b**) Folded groove structure of the abalone (reproduced with permission from [43,44], Zhang Yun et al., Copyright 2019, and Elsevier Ltd., Copyright 2009, respectively): (I) Images of the abalone’s pedal foot; (II) macroscopic view of fold grooves on the surface of the abalone’s gastropod; (III) cross−section of the foot pedal. The surface of its foot can contract and expand through the fold freely. (**c**) Morphology of suction cups of the medicinal leech (reproduced with permission from [39,40], the Royal Society, Copyright 2016, and Wiley Periodicals LLC., Copyright 2021, respectively). In image (**c**), (I, II), and (V, VI) are the light microscopy and SEM images of the anterior sucker (III) and posterior sucker (IV) of H. verbena, respectively. (**d**) The remora and its attachment microstructure (reproduced with permission from [45,46], The Company of Biologists Ltd., Copyright 2015, and Michael Beckert, Copyright 2015, respectively): (I) Macrograph of the suction pad of live remora. The arrows show its fleshy lip and lamellar compartments, respectively; (II, III) photograph of the detached suction pad with highlighted structural features. The arrows show its lamellar compartments and spinules, respectively; (IV) SEM image of spinules protruding from the suction disc. (**e**) Attachment structures of Epeorus assimilis larva (reproduced with permission from [47], The Company of Biologists Ltd., Copyright 2010): (I) Its ventral view; (II) the claw of its first leg; (III) setae of the pads on its ventral side; (IV) areas with spiky acanthae on the lateral parts of its abdominal sternites. (**f**) A teleost and its attachment microstructure (reproduced with permission from [48], Elsevier Ltd., Copyright 2006): (I) The arrowheads of a teleost. They are from a specimen of 12 cm in length, scale bar: 4 mm; (II) the epidermal cells with hexagonal outlines. They can be seen at the base of the spines. (**g**) The suckermouth catfish and mastoid microstructure (reproduced with permission from [49], American Scientific Publishers, Copyright 2014): (I) Its mouth in ventral view. It can show the dermal papillae covering the lip tissue, scale bar: 10 mm. The arrows show its lip papillae, upper jaw, lower jaw, and lower lip, respectively; (II) the enlarged image of its fungiform papilla. (**h**) Gyrinocheilus aymonieri and its rasp microstructure (reproduced with permission from [50], The Zoological Society of London, Copyright 1986): (I, II) Its oral sucker and a close-up of the posterior rasps. The arrows show its anterior rasp, posterior rasp, invaginations of the lower lip, dentary, median bar, and horny hooks, respectively. (**i**) The tooth and setae in the mouth sucker of the parasitic lamprey (reproduced with permission from [51,52], Springer−Verlag, Copyright 1983, and Kluwer Academic Publishers, Copyright 2003, respectively). (I) Its open mouth can act as a sucker for attachment; (II) in this image, the arrows show its anterior lingual tooth, posterior lingual tooth, supraorbital tooth, intraoral tooth, lateral tooth, upper labial tooth, lower labial tooth, marginal tooth, and seta in the open mouth, respectively. (**j**) (I, II) are a male diving beetle and the stereoscopic micrograph of its anterior suckers (reproduced with permission from [53], the Royal Society, Copyright 2014). The arrows show its larger sucker, little sucker, middle sucker, and seta, respectively.

For example, the octopus has unique soft suction cup structures of the tentacle [24], whose suction cups are mainly composed of the funnel body and the acetabulum (Figure 1a) [25,26,27]. The funnel body is partially surrounded by a ridged structure covered with grooves and folds. The attachment force of the suckers of the octopus can reach more than 20 times its weight, and its strong attachment ability is inseparable from the gully-like structures of its funnel body [28,29,30]. In the whole process of sucker attachment, the ridge and groove of the funnel body provide good waterproof sealing, which is the key to ensure the stable attachment of the octopus sucker [31]. Furthermore, with appropriate neuromuscular control, the funnel grooves and surrounding mucus can work together to further improve the non-slip and waterproof seal of the sucker [32]. The leech is a freshwater annelid with a gully-like structure [33,34,35]. Each leech has two suckers at the front and back, and its posterior sucker is larger than the anterior sucker on its head, which together help it to achieve attachment to the surface of the host and inchworm crawling [36,37,38]. As shown in Figure 1c, although the anterior and posterior suckers of the leech are different in morphology and structure, there are many glands, gully-like folds, and small pit-like structures on both surfaces [39,40]. The anterior sucker of the leech has a pore structure, and there are many irregular folds covered with mucus on its surface, which can be used for attachment and blood-sucking. At the same time, its posterior suction cup is a highly deformable complex composed of epidermis, smooth muscle, and collagen, which can exhibit excellent adaptability to different contact surfaces [33,41]. The gully-like structures on the suction cups of the leech can provide stable negative pressure and increase friction with the contacting surfaces to avoid slippage during the attachment process. In addition, there are also a large number of micron-scale pit structures on the surface of its suction cups, which can evenly distribute the mucus on the fold on the suction cup surfaces to form a wet seal. Studies have shown that surfaces with groove-like skin folds and microscale and nanoscale pit structures have better attachment properties than flat surfaces [39,42].

**Figure 2:**
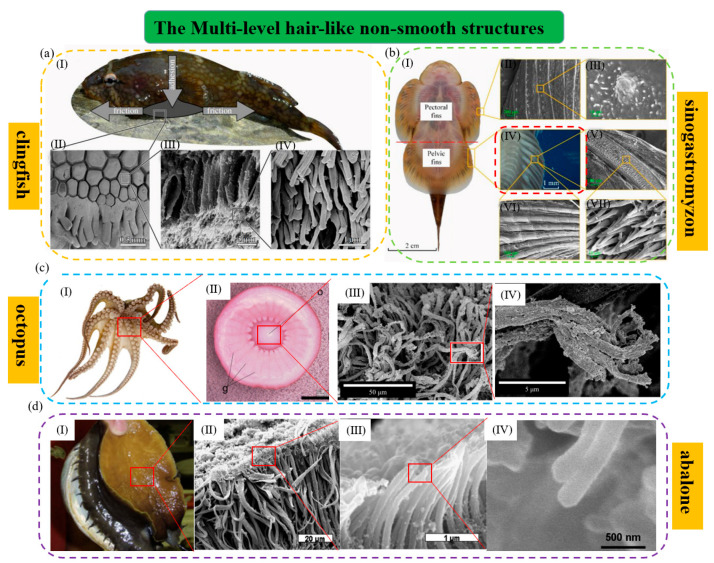
The multi−level hair−like structures of typical aquatic organisms. (**a**) The suction cup of the clingfish (reproduced with permission from [83], the Royal Society, Copyright 2013): (I) The clingfish attaches to a rock surface; (II) SEM image of the ventral surface of the attached suction cup. It shows the tiled papillae covered in the fimbriate edge and the mucus. Papillae can be found at the edge of the suction cup and other points of the surface; (III) SEM image of a papilla. The papilla consists of lots of rods that are subdivided apically into many fine filaments; (IV) SEM image of the filaments on the tips of the rod−like structures. (**b**) Microstructural characterization of *Sinogastromyzon szechuanensis* observed with SEM (reproduced with permission from [15], Cong Qian et al., Copyright 2021): (I) The suction cup of *Sinogastromyzon szechuanensis*; (II) the spinules. They are distributed on the longitudinal ribs of the pectoral fins; (III) the micromorphology of the pelvic fins; (IV) a layer of densely distributed trichomes on the surface of the pelvic fin; (V) the magnification of the spinules; (VI) the trichome covering the longitudinal rib surface of the pelvic fins; (VII) magnification of the trichome in (V). (**c**) The octopus and its hairy multi−level structure (reproduced with permission from [11,84], IOP Publishing Ltd., Copyright 2015, and Beilstein−Institut, Copyright 2014, respectively): (I) The octopus and its suction cups; (II) the frontal section of the infundibulum of the octopus and the externally visible portion of its sucker. The scale bar indicates 5 mm; (III, IV) magnified view of hairy multi-level microstructure of surface on the acetabular protuberance. (**d**) The abalone and its hairy multi−level structure (reproduced with permission from [44,54], Elsevier Ltd., Copyright 2009, and Elsevier Ltd., Copyright 2017, respectively): (I) An abalone can be attached to a human finger only using a fraction of its abdominal foot; (II–IV) they are SEM images which show the detailed nature of foot surface with fibers terminating in nanofibrils.

Additionally, some aquatic organisms can use gully-like structures to attach and act as the driving device for their movement. For example, the abalone can use its gastropod for attachment and activity. Its attachment capacity is powerful and can reach about 200–300 times its body weight [54,55]. Its attachment force comes from a wide range of sources, in which negative pressure vacuum attachment plays a decisive role in the entire attachment process [56]. As shown in Figure 1b, the gastropod of the abalone is wide, flat, and hypertrophic, and can be divided into three layers. The inner surface accounts for most of the gastropod area and many striped fold structures are located here. The folded structures provide free expansion and contraction of the surface of the abalone gastropod and can be an important source of vacuum suction and driving force that allows the abalone to attach and move freely [57]. Differently, *Sinogastromyzon szechuanensis* has a strip-like groove structure that mainly consists of lips, overlapping pectoral fins, and fused pelvic fins [58]. As shown in Figure 2b(IV), the external rays of the pectoral and pelvic fins have a striped appearance with prominent lateral ridges and strip-like grooves in an alternating fashion [15]. Upon further observation, it is found that the strip grooves are arranged between the transverse ridges, and there are no small thorn-like structures inside [59]. Strip grooves are conducive to rapid drainage and effective sealing during attachment. They can help it to form a vacuum attachment area at the groove quickly and help to improve its attachment capacity.

To sum up, the reasons why the gully-like structures can help aquatic organisms improve their attachment capacity are mainly reflected as follows [60,61]: (1) The gully-like structures help to achieve the peripheral extension of the biological soft suction cups and increase the contact with the surfaces of objects. (2) The softer structures, such as wrinkles, pits, and grooves, can help the suction cups to achieve space compensation when they contact irregular contacting surfaces, and the increasing friction between the contacting surfaces makes the sucker have higher attachment performance. In addition, the surface folds of some organisms (such as the abalone) can also become an important driver for their free movement.

#### 2.1.2. The Spinous (Barb)-like Structures

Mechanical interlocking and friction can enhance the attachment capacity of aquatic organisms by matching them with micro/nanostructures on irregular surfaces. Therefore, some aquatic organisms can achieve effective underwater attachment with the help of unique structures such as styli, spikes, and small hooks.

For example, the remora can attach to various fast-moving host organisms and marine vessels, and hitch firmly and reversibly with the help of its unique dorsal suction cup [62,63,64,65,66] (Figure 1d). The sucker of the remora is mainly composed of the lip, compartment, and comb-tooth lamella. It is important to mention that thousands of small styli distributed on the comb-tooth sheet play an important role in achieving a firm and fast attachment–detachment switching on the mobile hosts by increasing friction [67,68]. The styli are small mineralized tooth-like protrusions with a spinel geometry. They are mainly composed of micron-sized stiff hairs that protrude from individual comb-tooth lamellae and terminate as sharp cone-shaped points of varying shapes [69]. Studies have shown that a style can significantly increase the resistance between the sucker of the remora and the hosts’ uneven local surfaces to sliding, thereby enhancing its friction and shearing strength [70,71]. As the comb lamellae rotate in a direction, the style can withstand exposure to the large shearing force caused by the movement and avoid being thrown off by the hosts. Studies have shown that the adaptability of a style to different attachment surfaces is one of the important reasons why the sucker of the remora can attach to a variety of marine organisms and hitch in the ocean [45]. In addition, as shown in Figure 1e, the mayfly larva possesses strong underwater attachment, which is inseparable from the claws of its first legs, the spine pad on the ventral margin of the gill lamellae, and the spikes on the abdominal sternum. The spine pad of the mayfly larva, covered with bristles, can increase its friction and seal on rough surfaces, and structures such as claws and spikes can make it hook tightly on the host surfaces and enhance its tangential friction [72,73,74,75]. As shown in Figure 2b(III), *Sinogastromyzon szechuanensis* can also use its conical spinules on the longitudinal ribs of the pectoral fins to improve its attachment capacity. In turbulent water flow, the interlocking between the spinules and the attachment surfaces can further help increase the friction with stones and resist the impact of the water flow. Furthermore, as shown in Figure 1f, a teleost with small hooks on the mandibular sheath can interlock, rub, and attach to irregular contact surfaces through a similar attachment mechanism [48].

In a word, the roles of the spinous (barb)-like structures in improving the attachment capacity of aquatic organisms are mainly manifested by increasing friction. These structures can be fastened to the uneven surfaces on the attached substrates to form tiny mechanical interlocks and increase the tangential friction to achieve their stable and reversible attachment to stationary and moving objects.

#### 2.1.3. Other Single-Level Non-Smooth Structures

In addition, organisms have also evolved many other single-level non-smooth structures to help them enhance their underwater attachment capacity, including papillary structures [49], horny rasps [17], single-level hair-like structures [76], microsucker groups [53], etc. These single-level non-smooth structures mainly enhance the attachment capacity of their suction cups by increasing friction and sealing.

The papillary structure effectively increases the friction between attachment organisms and the substrates. As shown in Figure 1g, the suckermouth catfish is a freshwater fish and it attaches by its mouth sucker. There are various shapes of papillary structures in its mouth sucker, which may help it attach to glass and other objects [77,78,79]. Interestingly, attachment and free breathing can be completed simultaneously when the suckermouth catfish is attached to a cylinder wall [78,80]. Some aquatic creatures use horny rasps similar to papillae to increase friction with attached objects. For example, there are two horny rasps on the inside of the lips of *Gyrinocheilus aymonieri*, each with several rows of regular small hooks, and the scrapers of the hooks, whose main structures are cellular connective tissue, are keratinized (Figure 1h). When *Gyrinocheilus aymonieri* is attached, its lips are everted, and the entire horny rasps contact the contacting surfaces to increase the friction coefficient of its attachment [50]. In addition, a hairy structure can also play the role of increasing friction and sealing. As shown in Figure 1i, there is a suction cup in the mouth of the lamprey, and lots of villi are found at the edge of the suction cup, where adjacent villi can be closely attached [51,52,81]. When there is a leaky channel on the attachment surface of the lamprey, the villi fold and seal the leaky channel immediately. Since the villi effectively fill and seal the grooves on the attachment surface, the air pressure leakage rate on the rough surfaces can be about one-third lower than that on the smooth surfaces [81]. In addition, as shown in Figure 1j, the tarsus of the forefoot and midfoot of the male diving beetle can expand and specialize into a microsucker group structure. The structure of the suction cup group consists of three types: One large suction cup, one middle suction cup arranged in parallel, and about 200 small suction cups densely arranged around [82]. These tiny suction cups of different sizes can provide negative vacuum pressure for attachment. In addition, due to the minimal pore size of its suckers, they can also be regarded as unique papillary structures that can increase friction.

In a word, different structures also play different roles in the attachment process. The papillary structure and horny rasp mainly enhance the attachment capacity by increasing friction with the contacting surface, while the single-level hair-like structure can not only increase friction but also help enhance the air tightness of the sucker. Additionally, the suction cup group can gather the vacuum suction force of multiple suckers, thus enhancing the attachment force.

### 2.2. The Multi-Level Hair-like Non-Smooth Structures of Aquatic Organisms and Their Attachment Mechanisms

The hair structures of attachment organisms are important guarantees to enhance the attachment force and maintain long-term sealing [84,85]. For some aquatic organisms, many multi-level structures have been found on the microscale, and even nanoscale, in their hair structures, which may add important weight to the significant enhancement of their attachment capacity. Unlike multi-level hair-like structures of some terrestrial organisms such as geckos and beetles, most of the hair-like structures of aquatic attachment organisms do not have a spoon-like end structure that can generate van der Waals forces [86,87,88,89]. However, the attachment capacity of aquatic organisms is not affected by the combined action of multi-level hairs.

The clingfish is one of the representatives of organisms with the multi-level hair-like structure, whose attachment force can reach 80 to 250 times its weight. Of course, its high attachment capacity is inseparable from the protruding multi-level hair-like structure on the underside of its sucker edge [83,90]. As shown in Figure 2a, its papillary multi-level hair-like structure is composed of a large number of cylindrical fibers with a diameter of 4 μm and even smaller filaments with a diameter of about 0.4 μm on the tip of each cylindrical fiber [91]. Its multi-level hair-like structures can not only allow it to adjust the position to adapt to various irregular attachment surfaces and resist various axial disturbances and shearing forces, but also help it utilize hydrodynamic attachment and wet friction, reduce the suction cup’s sliding, and strengthen its sealing performance [92]. As shown in Figure 2c, the octopus also has multi-level hair-like structures whose shapes are slightly different from that of the clingfish. The surface of the acetabulum of the octopus is densely covered with a network of brush-like hair structures about 50 µm long and about 2 µm in diameter, each of which branches off at the tips into filaments about 5 µm long and about 0.3 µm in diameter [84]. These hair structures can also help the octopus enhance the sealing and attachment of its suction cups. By adopting a hierarchical peeling approach, researchers have evaluated that the group of hair structures can improve attachment performance by more than 30% at smaller peeling angles. Additionally, further studies have shown that in the presence of a layered structure, the first level contributes more to the increase in attachment, followed by the second level, and the attachment contribution becomes very small at the third level [93]. In addition, as shown in Figure 2b, cone-like multi-level hair-like structures have also been found on the pectoral and pelvic fin rays of *Sinogastromyzon szechuanensis* [94]. Similarly, the multi-level structure can not only form a supplement or buckle with the uneven contacting surfaces to increase friction but also help its suction cup to adapt to the contact surfaces with different surface morphologies and roughness to increase the sealing. This ensures the suction cup maintains strong attachment capacity on various contacting surfaces [95].

However, different from the above aquatic organisms, some organisms such as the abalone have spherical structures in the filamentous fibrous end of the micron-scale multi-level hair-like structures (Figure 2d), which is similar to terrestrial creatures like geckos and insects. That may be the reason why the sucker of the abalone can produce van der Waals force [44]. The multi-level hair-like structures of the abalone can make it fit nicely on the surface of rough objects, improve its lateral friction and sealing effect, and enhance its vacuum attachment. Thanks to the combined action of van der Waals force, capillary force, and mucus produced by the multi-level hair-like structures, the abalone has better attachment capacity than the clingfish and tree frog [55]. Additionally, similar hair structures of different shapes on the anterior tarsus joints of some male diving beetles’ forelimbs have been found. Approximately spatula-shaped or circular sucker-like structures are distributed at the end of the hair-like struts. These structures may help the male diving beetle attach to the female diving beetle’s elytra by using a combined suction and viscous resistance mechanism during underwater courtship and provide the male diving beetle with better attachment and control during locomotion and mating [53,82].

In conclusion, the high attachment capacity of aquatic organisms with multi-level hair-like structures is mainly due to their hierarchical attachment mechanisms consisting of suction and friction. Specifically, on the one hand, mucus is generally secreted around multi-level hairs, and the hairs, the mucus, and the water can form a high-performance attachment system [56,96]. In addition, the multi-level hair-like structures can increase the viscosity coefficient and shear resistance at the interface through combined action with mucus and water, thereby increasing friction and preventing the slippage and leakage of the suction cup edges. On the other hand, the multi-level hair-like structures are beneficial for the suction cups to adapt to contacting surfaces of different levels of roughness, which is more conducive to increasing waterproof sealing and prolonging the attachment time.

However, it needs to be emphasized that aquatic organisms’ environments are relatively complex and unpredictable. Therefore, to enhance their attachment capacity, many aquatic organisms have evolved various non-smooth structures, and they can complete the attachment tasks by configuring different non-smooth surface structures rationally. For example, gully-like structures can help realize vacuum attachment creation for the octopus and the abalone. Their multi-level hair-like structures and secreted mucus can increase friction and waterproof sealing properties [12,44]. For *Sinogastromyzon szechuanensis*, its strip-like extended groove-like mechanisms, small thorn-like structures, and multi-level hair-like structures can help it achieve the goal of vacuum attachment, increasing friction, and enhancing sealing, respectively [15]. The remora’s soft lip plate, compartment composed of comb-tooth sheets, and multiple comb-tooth sheets can be used for sealing, creating negative vacuum pressure, and enhancing friction to resist external shear forces [97,98]. Moreover, the small suction cup group structure and the multi-level hair-like structure of the male diving beetle can help it achieve vacuum attachment and enhance its attachment performance, respectively [82]. However, there are still no exact answers to how to control and in what proportion to use these various non-smooth structures to complete the whole attachment process, and researchers are still exploring these questions.

## 3. Basic Models and Force Testing Experiments of the Underwater Attachment of Aquatic Biological Suckers

To better explore the in-depth mechanisms of aquatic biological attachment and lay a good foundation for developing new biomimetic attachment equipment, the basic model of underwater attachment of aquatic natural suction cups and testing experiments of marine biological attachment force have been introduced by researchers in recent years. A summary of the above two aspects is presented to deepen the understanding of the underlying mechanisms of the attachment surfaces of aquatic suckers.

As shown in Table 1, it is found that most aquatic organisms rely on suction, capillary force, viscous force, mechanical interlocking, and a small amount of van der Waals force for attachment in the wet environment of water. However, in the underwater environment, since van der Waals force and capillary force are significantly weakened, only negative pressure suction and mechanical interlocking play a significant role. Therefore, in this section, only the basic models of suction and friction commonly used in the underwater attachment of aquatic organisms are described.

### 3.1. Basic Models of Underwater Attachment of Aquatic Organisms

#### 3.1.1. Suction

Suction is the most important way and means to complete the underwater attachment tasks for most aquatic attachment organisms. Although the shapes of the suction cup surfaces of various attachment aquatic organisms are different, their suction attachment mechanism is the same. That is to say, a closed attachment surface and a pressure difference between the inside and outside are formed. In general, the basic expression of the model for suction can be summarized as [83,91]:

(1) $$F=A\cdot \Delta P=\pi R^{2}\cdot \Delta p$$

where *F* is the attachment force, *A* and *R* are the area and radius of the suction cup, respectively, and Δ*P* is the pressure difference between the outside and inside of the suction cup. This model generally applies to aquatic organisms that rely on suction to attach underwater, such as octopuses, abalones, clingfish, etc.

For example, an artificial octopus-inspired suction cup with a microdome has been developed. When the peeling of its elastic film is assumed to be axisymmetric, its capillary-assisted suction expression concerning preload in wet attachment can be expressed as [99]:

(2) $$\sigma_{w}≅-2.145\Delta P\sqrt[{3}]{F_{P}}\cdot \left(\sqrt[{3}]{\frac{\left(1-v^{2}\right)}{E}}\right)\left(\frac{Rkn\left(2R+l\right)}{\sqrt[{3}]{tr_{0}}}\right)$$

where *σ* is the wet attachment force, *ΔP* is the pressure difference compared to the outside. Inside the suction cup, *F_p_* is the external preload force, and *v* is the material Poisson’s ratio. *E* is the elastic modulus of the material, *R* is the contact radius of the suction cup, *k* is the yield of the suction cup, *n* is the number of suction cups per unit area, *t* is the cavity depth of the suction cup, *r*_0_ is the radius of the artificial suction cup dome, and *l* is the circle distance from the tip end to the contact surface.

#### 3.1.2. Interlocking Friction

Friction is the tangential resistance created by one surface moving over another. When the surface of the natural suction cup is completely immersed in water, the surface tension is zero, and the capillary force does not affect the friction. Therefore, underwater friction by mechanical interlocking is usually reduced compared to wet friction [73]. However, aquatic attachment organisms may secrete a liquid or substance that alters the properties of the water to compensate for this friction loss. Generally speaking, the friction force is usually combined with other attachment mechanisms such as negative pressure attachment to improve the attachment capacity of organisms. The equation of the simplified model generally is [61]:

(3) $$F_{s}=\mu \cdot F$$

where *F_s_* is the tangential friction force, *μ* is the coefficient of kinetic friction, and *F* is the pressure applied vertically.

Researchers designed an interlocking adhesive based on the microstructure of a hydrogel, which works by mechanical interlocking friction. By using the force balance theory based on microhook arrays, the tangential friction force expression can be deduced, namely [100]:

(4) $$F_{s}=nF_{ext}\cos\left(\theta_{ext}\right)=n\left[F_{x}\cos\left(\frac{\pi }{2}-\theta_{m}\right)+F_{y}\cos\left(\theta_{m}\right)\right]$$

where *F_s_* is the tangential friction force, *n* is the inclination angle of the microhook, *θ_ext_* is the angle of the external force on the horizontal plane, *F_x_* and *F_y_* are the separation of the external force on the *x*-axis and *y*-axis, respectively, and *θ_m_* is the inclination angle of the microhook.

### 3.2. Force Testing Experiments and Related Research of Aquatic Biosorption

To better study the potential mechanisms of aquatic biological attachment, the attachment force testing experiments of aquatic biological suction cups (such as normal attachment force, tangential shearing force, or friction force) and the force measuring systems used by researchers in recent years are summarized. Generally speaking, according to the size of the attachment force, the attachment force test systems are mainly composed of force-measuring devices, including dynamometers or sensing devices of different specifications.

Various attachment force test systems have been designed for aquatic organisms with larger attachment forces. To sum up, attachment test systems which are composed of a standard dynamometer or a weighing sensor device with a more extensive range are generally used to test the attachment forces of their suction cups. The attachment forces typically include the normal and tangential forces. When measuring the normal attachment force, it is generally possible to connect the organism directly to the dynamometer for testing. When testing the shearing force (or friction force), a pulley is generally used to change the direction of the force.

**Figure 3:**
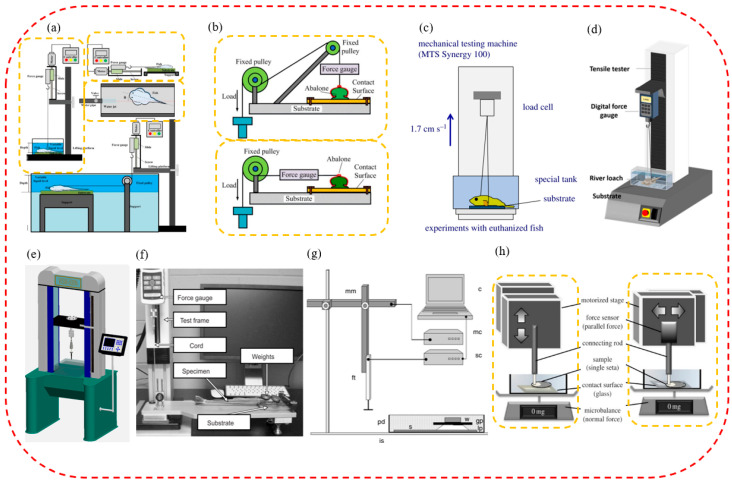
The test systems of attachment ability of suckers of aquatic organisms. (**a**) The experimental testbed for force measurement of *Guizhou gastromyzontidae*. It includes a normal attachment force test and shearing force out of water and underwater. (reproduced with permission from [101], Zou Jun et al., Copyright 2016) (**b**) The experimental testbed for the measurement of normal and shearing force of the abalone (reproduced with permission from [54], Elsevier Ltd., Copyright 2017). (**c**) The experimental design for attachment force measurements of clingfish (reproduced with permission from [91], The Company of Biologists Ltd., Copyright 2014). (**d**) The experimental design for attachment force measurements of the Pulin river loach (reproduced with permission from [94], Elsevier Ltd., Copyright 2017). (**e**) The experimental design for attachment force measurements of the leech as well as *Sinogastromyzon szechuanensis* (reproduced with permission from [15], Cong Qian et al., Copyright 2021). (**f**) The spinous friction test system of the remora (reproduced with permission from [45], The Company of Biologists Ltd., Copyright 2015). (**g**) The experimental design for attachment force measurements of Epeorus assimilis mayfly larvae (reproduced with permission from [47], The Company of Biologists Ltd., Copyright 2010). (**h**) The measurement design of the normal and shearing forces of a single seta for the diving beetle (reproduced with permission from [53], the Royal Society, Copyright 2014).

For example, as shown in Figure 3a, a digital force measurement system with a helical platform system was used when measuring the attachment force of a euthanized Pulin river loach. The system mainly included a lifting platform, HBO HF-50 dynamometer, motor, control switch, and optional pulley [101]. When the dynamometer pulled the Pulin river loach at a speed of 13.5 mm/s, the normal attachment force and tangential shearing force were measured on different substrates underwater and on other substrates. By the same token, as shown in Figure 3b, researchers used a similar attachment testing system when measuring the abalone’s normal and shearing bond strengths on different substrates both underwater and out of the water. The test system mainly included a fixed plate, a dynamometer (DS2-1000N), a pulley, and a pull rope. In the test work, homemade three-claw steel pliers clamped the abalone shell and connected it to a cable wrapped around a fixed pulley. The normal attachment force and tangential shearing force of the abalone on different contact surfaces were measured by a dynamometer. In addition, as shown in Figure 3f, the friction coefficient of the different-shaped spines of the remora on other contacting surfaces was measured [45]. The test system consisted of a lightweight and flexible pull cord which was attached to the Mark-10M520 dynamometer via a low-friction pulley. The test stand controlled the displacement of the pressure gauge, and the coefficient of friction was calculated by dividing the pressure gauge reading by the weight of the specimen.

Additionally, load cells were used to measure the attachment force in some force-measuring systems. As shown in Figure 3c, an attachment force test system was used with the sucker of the clingfish, which included a load cell (range 500 N), an MTS-type material test system, and a water tank with a replaceable surface [91]. While the crosshead was moving at a constant speed of 1 m/min, the researchers recorded the force continuously at 500 Hz and chose to analyze the highest pull-off force in each suction cup surface test. To evaluate the attachment performance of the Pulin river loach, a single-column universal test system was used in tensile mode (Figure 3d) [94]. An SH-100N digital dynamometer was connected to the universal testing system to measure the pull-off force more accurately. Additionally, the attachment capacity of suction cups of a leech on different contacting surfaces was also measured (Figure 3e). The equipment mainly included a double-screw column structure, a force sensor, a hooking device, a displayer, and a test substrate with different roughnesses. In addition, this equipment was also used to test the attachment force of *Sinogastromyzon szechuanensis* [15].

When measuring the attachment force of suction cups of aquatic organisms with minimal attachment force (such as a few micronewtons or less), a test device composed of a high-sensitivity microelectromechanical balance system was generally used. For example, an attachment test system was designed to measure the friction of the seat pads of mayfly larvae under different loading conditions, as shown in Figure 3g [47]. The system mainly included a computer, a motorized micromanipulator, a motor, a force sensor, a Petri dish, and a fixed plate. Friction measurements were performed with a combination of the constant motion of a motorized micromanipulator, and load cell force transducers completed the attachment force. Primarily, the larval gill seta pads’ friction was measured under three normal loads and four substrates. For example, the attachment test system measured a single circular or spatular seta’s normal attachment and tangential shearing force (Figure 3h) [53]. The attachment measurement system consisted of a stage, a force sensor, a connecting rod, and a microbalance. The normal attachment force was measured by a microbalance, while a force transducer was used to measure the horizontal shearing resistance.

In conclusion, researchers can more quantitatively explore the potential attachment mechanisms of aquatic organisms by measuring the attachment force (including normal pull-off force, tangential shearing force, etc.) of aquatic biological suckers on different contacting surfaces. This will lay a good foundation for the deep research of their attachment mechanisms and the development of advanced attachment equipment.

## 4. Research Status and Potential Application Fields of Biomimetic Attachment Equipment Inspired by Aquatic Organisms

The mysterious and excellent attachment ability of aquatic organisms can not only inspire ideas of researchers but also further open up the design path of advanced attachment equipment. With the deepening research on the unique micro/nanostructures and attachment mechanisms of various aquatic biological attachment surfaces, advanced bionic attachment robots, robotic arms, suction cup accessories, and micro-suction cup patches have been gradually developed [102]. For example, new underwater suction robots are inspired by the remora, and various bionic robotic arms, suction cup accessories, suction cup patches, etc., are inspired by the octopus [103,104,105]. It is worth noting that these biomimetic attachment devices already have excellent attachment performance. Presently, bionic attachment technologies are mainly used in bionic robots, flexible grasping robotic arms, wearable sensing devices, medical patches, etc. They have also played an important role in many fields such as underwater transportation, wall-climbing monitoring, flexible sensing, and medical treatments.

### 4.1. The Bionic Attachment Robots

At present, the robot industry is booming, and its applications are gradually transforming from industrial manufacturing to medicine and healthcare, aerospace technology, and deep-sea exploration. In recent years, robots with attachment functions have been developed gradually. They have been used in complex and extreme environments that humans cannot access to perform tasks such as monitoring, transportation, and exploration in the sea [106,107]. Nowadays, the principle and technology of bionic attachment are known and applied, and they have been applied to advanced machinery and equipment. The most representative examples are bionic soft attachment robots and bionic wall-climbing robots.

#### 4.1.1. The Bionic Soft Attachment Robots

The soft robot is a rapidly developing field in robotics. Compared with traditional rigid robots, soft robots have good flexibility, strong adaptability, and shock absorption. They have been widely used in human–computer interaction, scientific exploration, medical treatment, etc. [108,109]. Among soft robots, soft attachment robots with an increasingly wide range of applications have been playing an important role in production, packaging, transportation, and many other aspects. Compared with operation on land, bionic soft attachment robots are more complicated and challenging to operate in complex underwater environments. With the increasing demand for attachment robots underwater, developing high-efficiency, multi-functional, and intelligently responsive soft attachment equipment has become an urgent problem. In recent years, inspired by aquatic attachment experts such as the octopus, the remora, and the clingfish, advanced soft attachment robots with higher flexibility and adaptability have been gradually entering people’s field of vision.

**Figure 4:**
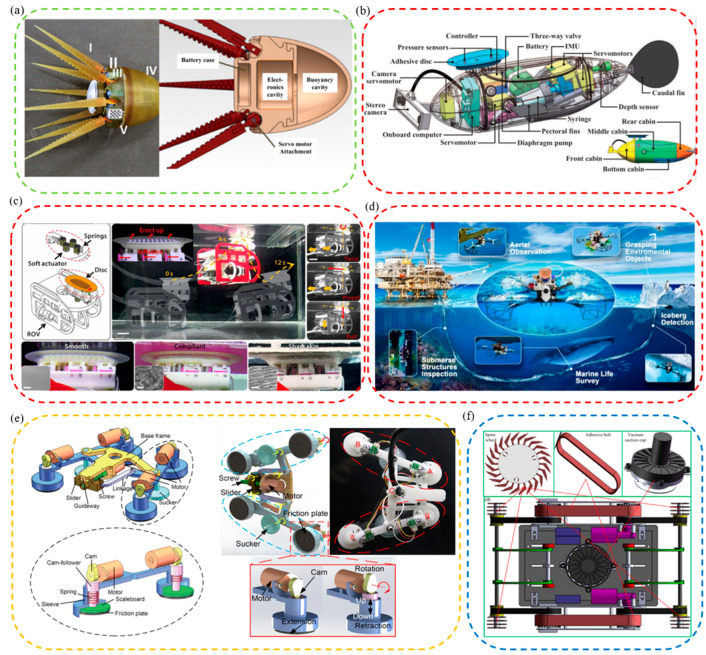
Bionic robots inspired by aquatic organisms. (**a**) A compliant eight-arm robotic prototype with PU arms (reproduced with permission from [110], IOP Publishing Ltd., Copyright 2015). (**b**) A mechatronic design of a robotic remora (reproduced with permission from [111], IEEE, Copyright 2021). (**c**) A biomimetic suction disc inspired by the remora. It can hitchhike underwater (reproduced with permission from [21], Wang Yueping et al., Copyright 2017). (**d**) A depiction of the mission profile and design of an aerial−aquatic hitchhiking robot. It can cross water and air and attach robustly to a wide variety of surfaces and perform long-term missions (such as monitoring) in natural environments (reproduced with permission from [22], Li Lei et al., Copyright 2022). (**e**) A rock-climbing fish-inspired robot crawling with load bidirectionally (reproduced with permission from [23], Zhejiang University Press, Copyright 2022). (**f**) A biomimetic motion and wall−climbing robot model (reproduced with permission from [112], Cambridge University Press, Copyright 2020).

For example, an octopus-like flexible arm robot was developed by Sfakiotakis et al. to give the attachment equipment better multi-arm maneuverability and the ability to grasp the target simultaneously (Figure 4a) [110]. This robot with a compliant platform and eight compliant arms could move forward, backward, advance, and steer underwater like an octopus. In addition, to allow the attachment mechanism to ride freely underwater with low energy consumption, a variety of underwater attachment robots that imitated the remora were designed. For example, a fin-driven underwater robot free-riding system was developed by Zhang Pengfei et al. (Figure 4b) [111]. It mainly included a front cabin, middle cabin, rear cabin, and bottom cabin. Specifically, the robot had the characteristics of strong maneuverability, strong perception, and reversible and long-term attachment, and could be used for the design of long-endurance robotic fish and mother–child multi-robot systems in the future.

Additionally, a lot of research had been carried out by Wen Li et al. to enable attachment robots to have stronger attachment on smooth, rough, and supple stationary and moving surfaces, and an underwater soft suction cup robot imitating the remora was developed (Figure 4c) [21]. The robot consisted of a soft lip and continuous overlapping sheets of composite material, on which a linear array of rigid carbon fiber needles was arranged. The research and development work of robots revealed the attachment mechanism of the remora and provided new ideas for future low-power underwater bionic soft robots and underwater attachment devices. However, suction cup robots still had the key problems of small moving range and insufficient maneuverability. Based on the above research, researchers continued to tackle key problems. Finally, they discovered a new mechanism-redundant attachment mechanism: A suction cup of a remora could attach to complex surfaces and achieve long-term attachment. As a result, a new robot based on redundant attachment that could swim, fly, and hitchhike across media was born [22]. As shown in Figure 4d, compared with the previously designed one, the new bionic remora suction cup robot could operate underwater and in air. It could also attach to various complex surfaces more efficiently for a long time and with low power consumption to perform long-term tasks. In addition, a foldable propeller could be folded underwater adaptively and unfolded in air passively so that the bionic robot could switch between water/air media more stably, continuously, and quickly. The attachment robot could carry out its job in many applications, including cross-media grasping, long-term underwater and aerial observations, marine biological surveys, environmental iceberg detection, etc.

#### 4.1.2. The Bionic Wall-Climbing Robots

Wall-climbing robots are unique robots that can work in a complex wall environment and carry specific tools to complete specific tasks [113]. They can replace humans to complete tasks such as ship rust removal and spraying, outer surface cleaning of high-rise buildings, civil engineering inspections, and other important tasks [114,115]. Suction cups that are attached by negative vacuum pressure are adopted as climbing feet by most wall-climbing robots to create better attachment and climbing ability [116]. However, when a wall-climbing robot uses negative pressure attachment to climb on rough, wet, or other complex walls, it may slip or fall due to unstable attachment pressure. In response to this phenomenon, some bionic wall-climbing robots with better attachment capacity and broader applicability have been designed by studying and imitating the attachment mechanisms of some aquatic organisms.

For example, *Beaufortia kweichowensi* can freely and energy-efficiently climb in two directions on slippery rocks with a non-detaching, low-resistance attachment method [95,101]. Inspired by it, a loadable bidirectional crawling bionic robot driven by linkage drive was developed successfully (Figure 4e) [23]. The robot included front and rear anisotropic attachment assemblies, each consisting of a commercial suction cup and two retractable variable friction modules. During its movement, power was provided by the oscillating drive module, suction was provided by suction cups, and additional friction for its suction motion was provided by the variable friction module. This bionic robot could successfully realize bidirectional crawling on horizontal, vertical, and inverted surfaces.

Generally speaking, the climbing area’s material greatly influences whether the climbing robot can be firmly attached and climb smoothly. Therefore, it is essential to improve further the stable climbing ability of the wall-climbing robots on various materials. Following flies’ and the clingfish’s motion and attachment principles, bionic flexible spine wheels, adhesive tapes, and adaptive eddy current suction cups were developed. Then, a new bionic wall-climbing robot was further proposed (Figure 4f) [112]. Experiments showed that the robot could crawl freely on various materials such as vertical surfaces, wooden surfaces, ceiling surfaces, etc., and its adaptability and climbing speed was also improved.

### 4.2. The Bionic Flexible Grasping Robotic Arms

Bionic flexible grasping robot arms can imitate some action functions of the hand and arm and can be used to replace people to complete many tasks. In recent years, they have been widely used in industrial, medical, and scientific exploration due to their excellent flexibility and adaptability [105,117]. For instance, they can be used to grab and transport fragile or rough objects efficiently and non-destructively and help people complete repetitive tasks such as routine packaging and transportation [118,119]. Unfortunately, for the current grasping robotic arms, it is still quite challenging to complete a series of operations such as retrieval, grasping, and conveying in confined, narrow, and complex environments [120].

In response to the above problems, inspired by the suction cup arms of ordinary octopuses and glowing sucker octopuses, a variety of bionic flexible grasping robotic arms that can be used for grasping objects in narrow and complex environments have been developed [121]. In general, bionic flexible grasping robotic arms can be divided into gripping and winding types according to their different grabbing methods.

**Figure 5:**
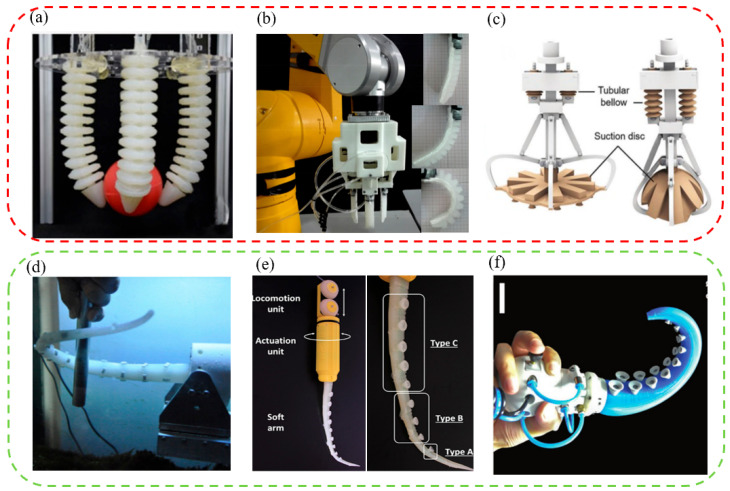
The bionic flexible grasping manipulators. (**a**) An adaptable grasping soft robotic arm which is actuated pneumatically (reproduced with permission from [122], The Chinese Society of Theoretical and Applied Mechanics, Copyright 2018). (**b**) A soft pneumatic dexterous gripper with convertible grasping modes (reproduced with permission from [123], Elsevier Ltd., Copyright 2019). (**c**) A glowing sucker octopus−inspired suction disc (reproduced with permission from [124], Wu Mingxin et al., Copyright 2022). (**d**) An artificial muscular hydrostat for developing an octopus−like robot (reproduced with permission from [125], Elsevier B.V., Copyright 2010). (**e**) A soft arm with suction cups inspired by an octopus for grasping tasks. It can be used in confined environments (reproduced with permission from [126], Barbara Mazzolai et al., Copyright 2019). (**f**) A soft tapered actuator with suckers for improved grasping inspired by octopus arms (reproduced with permission from [127], Mary Ann Liebert, Inc., Copyright 2020).

Various flexible gripping robotic arms were designed to achieve stable grasping of underwater objects. For example, imitating the flexible structure and unique motion mechanism of the ordinary octopus arms, a gripping adjustable robotic arm with multiple degrees of freedom was designed by Chen Zhe et al. (Figure 5a) [122]. It was a gripper-type robotic arm that could grab objects with four octopus-like components. Its arms were made of elastomeric materials that allowed large bends with variable stiffness in different directions and adjusted how it grasped things according to their shapes. By using a similar structure, a new soft pneumatic bionic manipulator composed of four soft fingers and an active suction cup was developed by Zhong Guoliang et al. (Figure 5b) [123]. The manipulator had four switchable grasping methods. Its soft fingers were silicone rubber with pleated channels, making the grasping process more flexible, safer, and including a wider grasping range. To further enhance the stability and applicability of grasping, inspired by the grasping ability of the sucker of the glowing sucker octopus, a soft gripper with adaptive and sensing capabilities was designed by Wu Mingxin et al. (Figure 5c) [124]. Compared with the above-mentioned mechanical arms, this machine adopted a combination of clamping and attachment, which had higher clamping stability and better adaptability. The soft gripper could grab and lift objects of different shapes and sizes, including irregular objects and out-of-range objects. In addition, the gripper could also perform sensory grasping in turbid water through devices such as eddy current sensors.

It is difficult to retrieve and grasp objects of unknown size in narrow, airtight, and slippery spaces stably. Therefore, various winding flexible grasping manipulators were developed to solve the above problems. For example, a robotic arm called an artificial muscle hydrostatic device was developed by imitating octopus arms (Figure 5d) [125]. It consisted of four longitudinal muscles and some parallel transverse muscles, which could imitate the octopus to complete complex movements such as elongation, shortening, bending, and straightening. In addition, to further improve the winding and clamping effect of the robotic arms, many small octopus-like suction cups with strong attachment capacity were designed on the inner surface of the robotic arms to increase the stability and reliability of gripping objects. For example, a soft robotic arm with a conical suction cup was designed by Barbara Mazzolai et al. (Figure 5e) [126]. The soft robotic arm consisted of tendons and fluid-active suction cups. The small suction cups were made of soft materials and enabled the manipulator to exhibit stronger manipulation capabilities in different media (such as air, water, and oil) confined environments, and with objects of unknown shapes and sizes. Additionally, the small suction cups could also help to retrieve and grab various objects with complex shapes in limited space. With a similar principle, a multi-functional conical gripper was designed and fabricated by Xie Zhexin et al. (Figure 5f) [127]. A larger and stronger conical brake was adopted, which made the gripper more efficient, flexible, and of good gripping ability. Through the combined action of suction and bending, the designed conical gripper required only one actuator to grasp various flat, curved, smooth, and rough objects in confined and narrow environments.

### 4.3. The Bionic Suction Cups and Micro-Suction Cup Patches That Can Be Used as Accessories for Bionic Attachment Robots

Presently, attachment devices are often integrated into robots as their attachment hands or climbing feet and help them complete stable attachment grasping and flexible attachment climbing. It is crucial to realize flexible movement further by making robots with fast sensing and stable attachment. Inspired by the octopus and other aquatic attachment organisms, a variety of bionic suction cups or micro-suction cup patches have been developed. They have good attachment effects and can be used as accessories for biomimetic flexible attachment robots and climbing robots.

#### 4.3.1. The Bionic Suction Cups That Can Be Used as Suction Accessories for Attachment Robots

**Figure 6:**
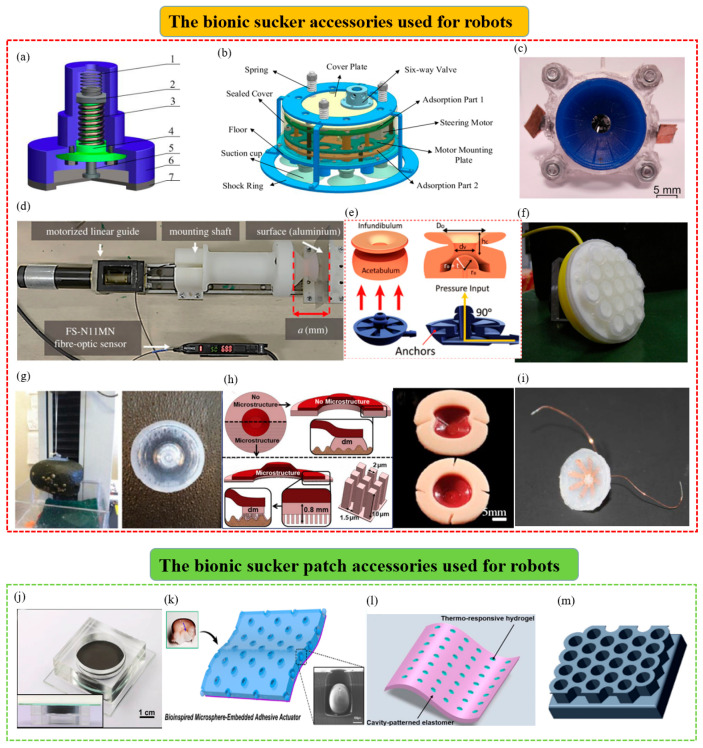
Bionic suction cups used as robot accessories. (**a**) A bioinspired miniature suction cup actuated by shape memory alloy (reproduced with permission from [131], SAGE PUBLICATIONS INC, Copyright 2009). (**b**) An octopus−inspired underwater vibration suction module (reproduced with permission from [132], IEEE, Copyright 2017). (**c**) An octopus−inspired suction cup with dielectric elastomer actuators (reproduced with permission from [128], IOP Publishing Ltd., Copyright 2014). (**d**) A sensing robotic anchoring module for attachment to the environment. It can be integrated into robots to enable or enhance many functions (reproduced with permission from [129], the Royal Society, Copyright 2017). (**e**) A bioinspired soft wet adhesion actuator with carbon nanotube−based strain sensors. It can be perceived electronically (reproduced with permission from [130], American Chemical Society, Copyright 2021). (**f**) An innovative suction cup inspired by the tube feet of sea urchins (reproduced with permission from [136], IEEE, Copyright 2012). (**g**) A suction cup inspired by northern clingfish attachment to rough surfaces (reproduced with permission from [134], the Royal Society, Copyright 2019). (**h**) A biomimetic hierarchical suction cup inspired by the attachment mechanisms of clingfish (reproduced with permission from [133], IOP Publishing Ltd., Copyright 2019). (**i**) A bionic micro−suction cup based on the structure and behavior of the suction cups of the leech. It can help a micro-medical robot stick to the inner wall of the gastrointestinal tract and perform a long-term test of peristalsis (reproduced with permission from [135], IEEE, Copyright 2014). (**j**) An octopus−inspired smart adhesive (reproduced with permission from [137], Wiley-VCH GmbH, Copyright 2020). (**k**) A bioinspired attachment architecture with embedded microspheres. It can be used as an electrothermally actuating transporting device of wet and dry pliable surfaces (reproduced with permission from [138], American Chemical Society, Copyright 2021). (**l**) A mobile magnetic pad that has fast light-switchable attachment capabilities (reproduced with permission from [139], IOP Publishing Ltd., Copyright 2021). (**m**) A honeycomb structure with a soft elastic film. It can partially cover the cavity of the honeycomb pattern (reproduced with permission from [140], Elsevier B.V., Copyright 2022).

(1) The octopus-inspired bionic end manipulators that can be applied to attachment robots.
  Based on the good underwater attachment effect of the octopus, various attachment manipulators suitable for underwater soft attachment robots were manufactured. For example, an octopus-inspired suction cup was developed by Follador et al. (Figure 6c) [128]. It was mainly composed of a driving device fixed by a plexiglass frame and a soft artificial funnel made of silicone. The actuation of the suction cup mimicked the acetabular radialis muscle of the octopus suction cup and was based on a dielectric elastomer actuator with an integrated actuation system. This suction cup system was especially suitable for soft robotic manipulators working in wet conditions.
  In addition, as shown in Figure 6d, based on the octopus’s perception system, a robot anchoring module with a sensing mechanism was also designed by Sareh et al. to enhance the attachment robot’s ability to move and maintain its position and manipulate objects [129]. This module could be used for robot motion planning and anchor fixation state measurement, and it quantified its ability to hold anchors under constant and variable vacuum pressure signals. Subsequently, inspired by the attachment, sensing, and decision-making functions of octopus suckers, a soft attachment actuator was developed by Lee Heon Joon et al. (Figure 6e) [130]. The strain sensor module was combined with the complex surface of the AOS to help it identify objects. The actuators were integrated with machine learning to help predict the weight of specific objects and determine their center of gravity for more stable and reliable attachment. To its credit, the soft adhesion actuator had the advantages of responsiveness, high durability, and repeatability.
(2) The octopus-inspired bionic suction cup attachment foot that can be applied to wall-climbing robots
  In order to further improve the attachment capacity of wall-climbing robots and realize their better climbing effect, researchers gained inspiration from the sucker of the octopus. They carried out bionic optimization designs for the attachment feet of wall-climbing robots. For example, a bionic micro-suction cup driven by SMA was designed by Hu Bingshan et al. [131] (Figure 6a). It comprised an SMA spring driver, a rigid edge, a guide element, a guide piece, and an elastic element. It was actuated by a biased unidirectional SMA actuator and could be used as an attachment mechanism for a miniature wall-climbing robot without an air pump. In addition, by simulating the muscle contraction and expansion of the octopus’s sucker, a suction module with good underwater vibration attachment capacity was designed by Chen Rui et al. (Figure 6b) [132]. It mainly included a vibration source mechanism, a release mechanism, a sealing mechanism, and a damping mechanism. The vibration source mechanism was a central crank-slider structure and two groups of suction cups could vibrate by controlling the assembly position of the two eccentric wheels. In addition, the release mechanism could complete the attachment and release of the machine on the wall by controlling the six-way valve. The mechanism might be used in the feet of underwater wall-climbing robots in the future.
(3) The bionic suction cup accessories inspired by other aquatic organisms that can be applied to attachment robots
  Many bionic suction cup accessories have been inspired by aquatic organisms other than the octopus and could be integrated with attachment equipment. For example, based on the influence mechanism of the papillary hair-like hierarchical structure on the surface of the sucker of the clingfish, a biomimetic pharynx sucker was designed by Sandoval et al. (Figure 6h) [133]. The suction cup with good adaptability on smooth and rough surfaces could be used for suction robots. In addition, a biomimetic fish sucker with a microstructure was designed by Petra Ditsche et al. (Figure 6g) [134]. It could attach to rough surfaces, non-planar geometries, and surfaces of fragile objects stably and could be used as a flexible attachment part of ROV manipulators. Inspired by the structure and performance of the suction cups of the leech, a miniature bionic suction cup was designed by Feng Huashan et al. (Figure 6i) [135]. It was powered by a petal-like ionomer metal composite (IPMC) and wrapped in harmless silicone rubber. It could be used as an attachment mechanism for a micro-medical robot to help it attach to the inner wall of the gastrointestinal tract for long-term peristalsis experiments. In addition, inspired by the adhesion mechanism of sea urchins, a suction cup that was suitable for both underwater crawling robots and manipulators was designed by Sadeghi et al. (Figure 6f). It combined a soft suction cup and chemical adhesive material and could have better attachment capacity on rough surfaces [136].

#### 4.3.2. The Bionic Micro-Suction Cup Patches That Can Be Used as Suction Accessories for Attachment Robots

As well as bionic suction cups, many wet bionic suction patches could also be applied to robots to ensure more precise and stable attachment in wet environments or on rough surfaces. Still taking the octopus as an example, inspired by its suction cup’s elastic energy storage mechanism, a new type of magnetically actuated smart adhesive was designed by Wang Suhao et al. (Figure 6j) [137]. The adhesive could actively control the deformation of the elastic membrane by an external magnetic field to produce cavity pressure-induced attachment. It had the advantages of fast switching, highly reversible adhesive strength, and long attachment time. It could be used as the suction hands of suction robots to grasp and release rigid and deformable substrates within a certain roughness and curvature range. Additionally, a simple electrothermally driven transporting device for microsphere-embedded suction cups (OMS) was designed by Sangyul Baik et al. (Figure 6k) [138]. The assembled actuator could expand and contract actively and induce switchable attachment and detachment reversibly. This biomimetic device could be integrated into programmable transporting devices, and smart medical grippers and soft robots could flexibly attach and release dry and wet thin objects. Afterward, an optically switchable attachment patch with magnetic field-driven motion was designed by Su Lin et al. (Figure 6l) [139]. It could drive movement without restraining and controlling the attachment and separation process of attachment patches and could be used in assembling high-performance electronic components in confined spaces or closed environments in robotics. Furthermore, as shown in Figure 6m, a honeycomb structure with a soft elastic membrane was designed and fabricated by Khan Muhammad Niaz et al. [140]. The honeycomb structure could offer sufficient support to maintain the stability of the microstructure, and sufficient flexibility to form strong attachment could be provided by the soft PDMS film in the pattern. This design could be integrated into a robot and could serve many fields more effectively, such as non-destructive handling, bionic wall climbing, space operations, etc.

### 4.4. The Micro-Suction Cup Patches for Wearable, Flexible Sensors

Wearable electronic flexible sensors are gradually recognized due to their high efficiency, lightweight, and good comfort advantages. According to the sensing principle, sensors can be divided into pressure sensors, humidity sensors, temperature sensors, biosensors, and chemical gas sensors [141]. They have been gradually applied to the long-term monitoring of physical, electrophysiological, or biochemical signals and environmental conditions [142,143]. Newly developed flexible sensors are mainly based on emerging flexible electronics and nanomaterials and multiple critical functions are integrated into a thin and flexible patch. It has become an effective method to improve the sensing performance of various sensors [144,145,146,147]. While this kind of attachment patch can make the flexible sensor attach to the skin, it is easily deformed or falls off as human skin is generally rough, moist, hairy, and stretchable [148,149]. Moreover, it also may cause skin contamination, damage, and infection risks due to the poor biocompatibility of the materials used on the skin [150,151,152]. In general, the tight attachment between the flexible sensor and the skin surface is crucial to obtaining accurate and stable signals. Therefore, to enhance the sensing performance of flexible sensors, close conformal contact of the flexible substrate with the target substrate (such as skin) is essential. So, it is required that the patch substrates meet various requirements such as lightness, water resistance, strong attachment, good flexibility, and stretchability [153,154,155,156,157]. This is the best way to realize the biocompatibility and close conformal contact between flexible sensors and human skin to design and develop new wearable flexible sensors [158,159,160]. In recent years, to improve the accuracy and long-term monitoring of body signals, researchers have been developing flexible bio-integrated devices with highly soft and irregularly shaped skin or organ surfaces for a close conformal fit to the human body [161,162]. The good news is that attempts to incorporate bionic architecture into bioelectronics are working, and flexible sensors have been integrated into reversible bioinspired dry/wet bionic micro-suction cups. For example, pushrod-shaped dry attachment patches inspired by reptiles such as geckos are tightly attached and adaptable to the skin [163,164,165]. However, their ability to attach to sweaty skin remains to be improved. Therefore, people began to focus on octopuses and other underwater organisms with strong attachment abilities. For example, various wet/dry bionic suction cup patches that can be used for wearable soft sensors have been developed by drawing inspiration from their unique attachment surface. They can be applied to electrophysiology, electroencephalograms, electromyography, and other health indicator monitoring methods successfully [166,167,168,169].

**Figure 7:**
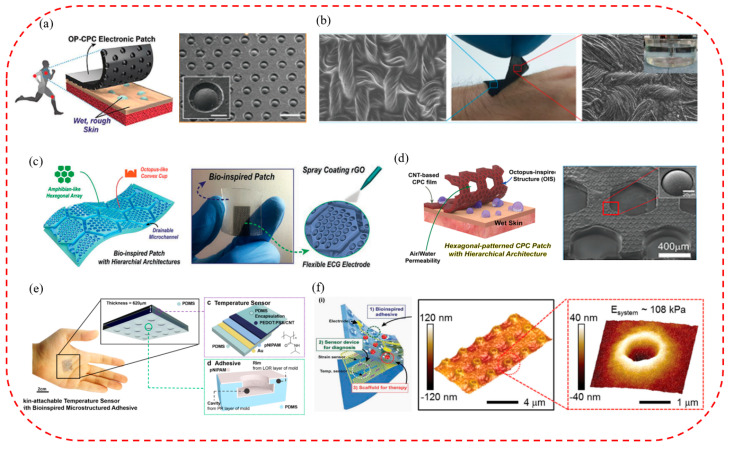
Micro−suction cup patches applied to wearable, flexible sensors. (**a**) Octopus−inspired carbon−based CPC electronics against dry/wet skin for biosignal monitoring (reproduced with permission from [172], WILEY−VCH Verlag GmbH & Co. KGaA, Weinheim, Germany, Copyright 2018). (**b**) A water−resistant and skin−attached wearable electronic. It uses a graphene fabric sensor with octopus−inspired micro−suction cups (reproduced with permission from [148], American Chemical Society, Copyright 2019). (**c**) A highly permeable bioinspired skin patch with conductive hierarchical hexagonal microstructures for omnidirectionally enhanced wet adhesion (reproduced with permission from [173], WILEY−VCH Verlag GmbH & Co. KGaA, Weinheim, Germany, Copyright 2019). (**d**) A high air/water−permeable hierarchical mesh architecture used for stretchable underwater electronic skin patches (reproduced with permission from [174], American Chemical Society, Copyright 2020). (**e**) A high−sensitivity bioinspired skin−attachable temperature sensor (reproduced with permission from [175], American Chemical Society, Copyright 2018). (**f**) A cephalopod−inspired electronic patch with bioinspired dry adhesives (reproduced with permission from [176], WILEY−VCH Verlag GmbH & Co. KGaA, Weinheim, Germany, Copyright 2015).

Due to the unique layering mechanism, the octopus has excellent attachment properties on dry, wet, and rough surfaces and is a critical biomimetic template for micro-suction cup patch fabrication for wearable, flexible sensors [170,171]. Therefore, by imitating the octopus, a variety of biomimetic micro-suction cup patch sensors that were waterproof, breathable, biocompatible, tightly attached, and reusable were developed. They could detect important monitoring indicators such as those of an electrocardiogram, body temperature, and blood pressure for a long time, even in wet conditions and on rough skin surfaces. For example, a conductive micro-suction cup patch imitating the dome-like structure of an octopus was developed by Chun Sungwoo et al. [172] (Figure 7a). In this patch, an octopus-like pattern was printed on a carbon-based conductive polymer composite film with good attachment and water repellency. It could be used as a wearable and skin-attachable sensor device for monitoring various biological signals by controlling carbon nanosheets in the polymer matrix. Subsequently, a scalable, facile, and low-cost skin-bonded graphene-coated fabric (GCF) sensor was developed by changing the material (Figure 7b) [148]. The sensor had a sensitive piezoresistive response to the dynamically and statically applied strain and a reliable response to repeated manipulations of applied pressure and strain. Due to the presence of OPS, GCF samples were tightly attached to the human skin in both wet and dry environments. It could monitor various human activities effectively, such as wrist pulse, electrocardiogram, body movement, and speech vibration. Inspired by the convex cup structure of an octopus’s suckers and the microchannel network in the toe pads of the tree frog, a skin patch that could be used to monitor ECG signals was also proposed by Kim Da Wan et al. [173] (Figure 7c). It had the advantages of high breathability, good drainage, strong attachment, and reusability. As the octopus-shaped convex cup structure was designed on the hexagonal structure’s upper surface, the suction cup’s attachment to the skin in sweaty and even running water conditions was further improved. In addition, based on the above research, the pattern of the patch contacting surface was further optimized by Min Hyeongho et al. [174], and a hexagonal grid pattern CPC patch was finally designed (Figure 7d). It could come into contact with rough and moist biological skin and have better drainage and attachment properties. In addition, it could not only detect the bending strain of fingers in wet and dry conditions sensitively but also measure ECG signals in an underwater environment stably.

In addition, the new bionic micro-suction cup patch sensors can also monitor other useful indicators of the human body effectively, such as temperature, and be used for continuous monitoring. By simulating the edge structure of the octopus, a resistive high-sensitivity flexible temperature sensor was fabricated (Figure 7e) [175]. It could be attached to and detached from the skin repeatedly and detect changes in skin temperature of 0.5 °C stably and accurately. The temperature sensor can be used in wearable medical and healthcare monitoring devices. To achieve a variety of monitoring functions, an ultrathin and stretchable micro-suction cup (MSC) dry adhesive was developed by Choi Moon Kee et al. (Figure 7f) [176]. It could be integrated with therapeutic nanoparticles, physiological sensors, and drug delivery actuators to create a novel glue-free diagnostic and therapeutic system. In addition, it can allow continuous monitoring of changes in vital signs, availability of drugs under various physiological or pathological conditions, controlled releasing and wireless communication with telehealthcare devices, etc.

### 4.5. The Smart Micro-Suction Cup Patches for Biomedical Therapies

The treatment of wounds has always been a common focus and problem. Compared with traditional treatments such as debridement, drainage, and suturing, skin attachment patches cause less damage and have multiple functions and low cost, and are becoming one of the ideal strategies for treating minor trauma [177]. However, despite the success of the skin-adhesive patch, it still has several limitations: (1) It is difficult to make close conformal contact with stretchable skin whose surface is covered with impurities such as hair, sweat, and debris; (2) the adhesive will cause particular irritation to the skin, which may cause allergic reactions such as redness, itching, etc.; (3) the adhesive will become dry after being placed for a long time, which will affect its attachment strength and duration on the skin. Some biomimetic-based multi-functional medical micro-suction patches have been developed through extensive research and exploration of various aquatic creatures. They have the advantages of good biocompatibility, effective attachment, and flexible applicability and can effectively solve the problems of allergy, wound infection, and shedding caused by traditional skin treatment patches. Additionally, they can play an important role in wound covering, dressing, hemostasis, and skin repair [178].

**Figure 8:**
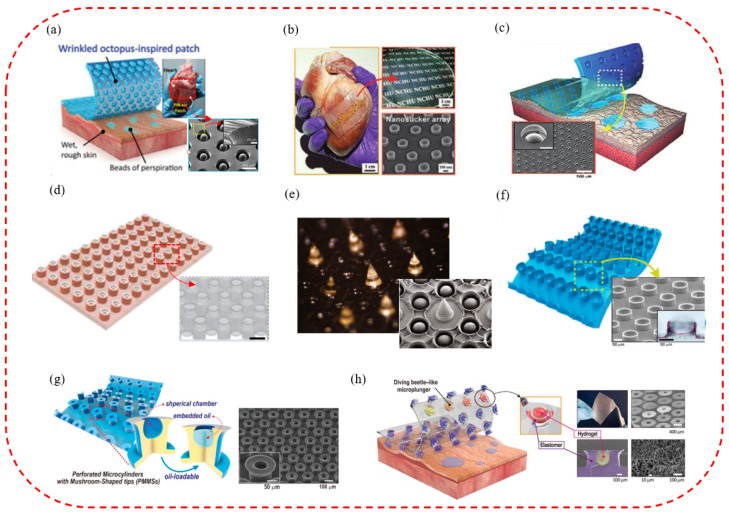
The bionic suction cup patches used for medical treatments. (**a**) An attachment patch inspired by the soft wrinkles of miniaturized 3D octopus suction cups (reproduced with permission from [180], American Chemical Society, Copyright 2019). (**b**) The assembly of nano−sucker arrays for dry/wet attachment inspired by the octopus (reproduced with permission from [181], American Chemical Society, Copyright 2017). (**c**) A wet−tolerant attachment patch inspired by protuberances of the sucker of the octopus (reproduced with permission from [182], NATURE PORTFOLIO, Copyright 2017). (**d**) A biocompatible wound−healing patch with individualized design and selective attachment by using tailorable patterns (reproduced with permission from [183], Huang Rongkang et al., Copyright 2021). (**e**) The bioinspired attached and antibacterial microneedles. They can be used for versatile transdermal drug delivery (reproduced with permission from [184], Zhang Xiaoxuan et al., Copyright 2020). (**f**) The highly adaptable and biocompatible attachment patches inspired by an octopus with meniscus−controlled unfolded 3D microchips for hairy skin and underwater surfaces (reproduced with permission from [170], Baik Sangyul et al., Copyright 2018). (**g**) The male diving beetle-inspired attached oil−loadable perforated microcylinders with mushroom-shaped tips (PMMSs). They can load oil in their spherical chambers (reproduced with permission from [185], Elsevier B.V., Copyright 2021). (**h**) The male diving beetle−inspired miniaturized plungers with rapid and reversible biofluid capturing for skin disease care based on machine learning (reproduced with permission from [186], Baik Sangyul et al., Copyright 2021).

The micro-suction cup patches coupled with the skin can not only monitor various important physiological and pathological signals of the human body stably and reliably but can also realize percutaneous medical treatment effectively. Studies have shown that biomimetic adhesives inspired by gecko feet or insect pads will lose the adhesive help of van der Waal forces in wet or aquatic environments [179]. As a representative of underwater attachment organisms, the octopus can produce a good attachment effect in wet and dry environments. It can be an important source of inspiration for bionic medical micro-suction cup patches. For example, inspired by the soft folds of octopus suckers, a biomimetic suction patch was designed by Baik Sangyul et al. (Figure 8a) [180]. The suction patch had high drainage and strong attachment to wet and dry human organs. Test results on wet organ surfaces in pigs indicated its good use in attachable wound dressings and bioelectronic devices. In addition, a simple and scalable self-assembled template technique for fabricating non-close-packed suction cups on PDMS substrates was developed by Chen Yingchu et al. [181]. As shown in Figure 8b, the templated nano-suction cup arrays could exhibit good attachment on wet and dry surfaces. Therefore, the technology could provide a platform for various medical applications such as wound hemostasis, care, and recovery.

Researchers have also been actively exploring solutions that can be used for rapid wound recovery and healing, and have developed a variety of related micro-suction cup patches. For example, a reversible dry/wet attachment system inspired by the dome-like protrusions in octopus suckers was proposed by Baik Sangyul et al. (Figure 8c) [182]. The system was developed to be simple to manufacture and non-polluting. It could exhibit strong, reversible, reproducible attachment to glass and rough skin surfaces in wet and dry conditions. Meanwhile, a PBS-loaded OIA patch could be applied to the wound surface and aided in wound healing. By adopting a strategy combining template replication and mask-guided lithography, a customizable wound patch with many advantages, such as selective attachment, good stretchability, and biocompatibility, was proposed by Huang Rongkang et al. (Figure 8d) [183]. The patch could be attached to different skin surfaces for personalized wound healing and was an ideal candidate for wound healing and other biomedical applications.

The microneedle suction cup patches recognized by the medical community have gradually joined the ranks of skin treatments. The patches have been paid attention to because of their painless, non-invasive, and efficient drug delivery method [187,188,189]. However, the practical application of microneedles in different epidermal environments and locations still suffered from the problems of low attachment and poor antibacterial activity, which greatly limited their development prospects [190]. Therefore, inspired by the attachment mechanism of octopus tentacles and mussel brachiopods, a graded microneedle sucker patch with multi-functional attachment and antibacterial capabilities was developed by Zhang Xiaoxuan et al. (Figure 8e) [184]. The microneedles were based on polydopamine hydrogel, and each microneedle was surrounded by a circle of cavities with a suction cup structure. The graded microneedles could attach to the skin for a long time in a dry and wet environment and help the skin repair itself. Furthermore, inspired by the rim and funnel body of octopus suckers, a highly adaptable and biocompatible suction cup patch was designed by Sangyul Baik et al. (Figure 8f) [170]. The patch featured meniscus-controlled non-foldable 3D microprobes that demonstrated stable attachment on wet, hairy, and rough skin without leaving chemical residues on the skin. The patch might raise good ideas for developing an attachable and conformable patch for wound healing and smart skin.

In addition, researchers have gained a lot of inspiration for the research and development of new micro-suction cup patches with different functions based on the special suction cup structure of diving beetles. For example, as shown in Figure 8g, inspired by the hairy structure of diving beetles, a reversibly attached patch with good adaptability and strong attachment was proposed by Song Jin Ho et al. [185]. The patch had a mushroom-shaped tip and an oil-filled spherical suction chamber that enhanced its attachment through its viscosity-assisted action on soft and moist organs, tissues, and skin. Additionally, it could be applied to the top surface of a surgical hand to minimize damage to organ surfaces such as diseased livers. The micro-suction cup patch might provide a new strategy for developing in vivo medical adhesives or intelligent robots. In addition, new research findings have shown that the pH value of the skin surface can be used as a clinical standard for medical and biochemical research. Therefore, human physiology can be monitored rapidly and effectively by capturing various biological fluids (such as sweat, saliva, tears, blood, etc.) and analyzing them effectively [191,192]. For example, inspired by the suction plunger in the male diving beetle setae, a bioinspired microplunger attachment patch was developed by Baik Sangyul et al. (Figure 8h) [186]. It had excellent biocompatibility properties, repeatability, and reversible attachment under dry/wet conditions. By embedding aquagel in microplugs, suction-assisted attachment could be enhanced, and a simple pH analysis could also be performed. Combined with technologies such as machine learning, the pH value could be quantified automatically to achieve rapid and effective monitoring of human information. Beyond that, the patch could be used to detect early signs of skin diseases such as acne, demonstrating reliable and effective treatment in vivo.

## 5. Conclusions and Perspectives

The colorful underwater world can always inspire human beings and provide infinite possibilities for designing and manufacturing advanced bionic attachment equipment. By studying the unique attachment structures of aquatic organisms, people have been inspired to design a variety of advanced bionic suction cups, and methods and ways for efficient attachment of advanced attachment equipment and materials have been expanded.

**Figure 9:**
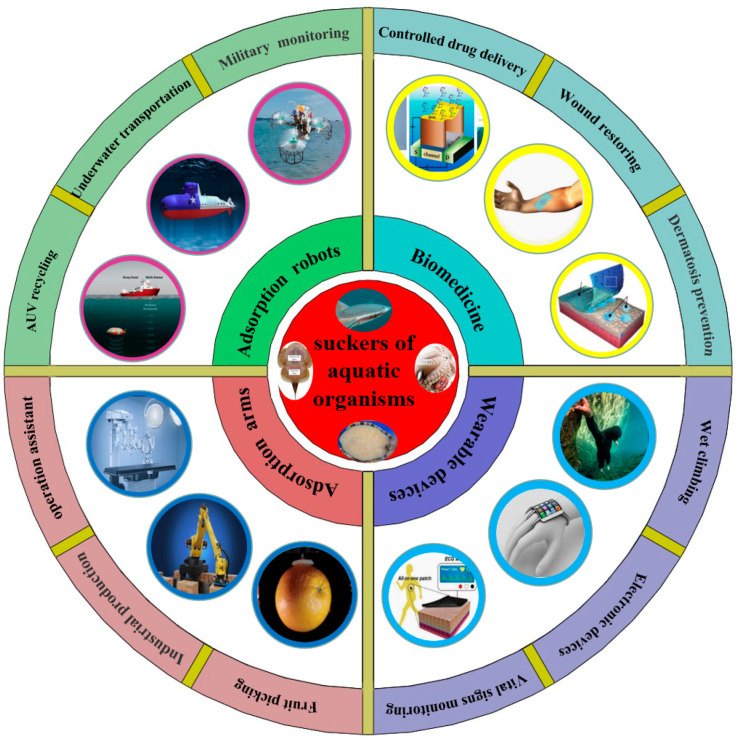
The application of biomimetic devices based on the attachment mechanism of suckers of aquatic organisms [61], which includes attachment robots (e.g., AUV recycling, underwater transportation, military monitoring, etc.), attachment arms (e.g., fruit picking, industrial production, operation assistant, etc.), biomedicine (e.g., controlled drug delivery, wound restoring, dermatosis prevention, etc.), and wearable devices (e.g., wet climbing, vital sign monitoring, electronic devices, etc.).

As shown in Figure 9, relevant bionic devices designed based on the attachment mechanism of aquatic biological suckers have made certain research achievements. They can be applied in intelligent attachment robots [21,22,23,110,111,112] (e.g., AUV recovery, underwater transportation, monitoring, etc.), mechanical attachment arms [122,123,124,125,126,127] (e.g., fruit picking for agricultural harvesting, manufacturing, packaging, transportation for industry, surgical robotic arm assistants for medical use, etc.), wearable devices [172,173,174,175,176] (e.g., wet climbing, vital sign monitoring, electronic sensing, etc.), and biomedical applications [180,181,182,183,184,185,186] (e.g., controlled drug delivery, wound recovery, dermatological prophylaxis, etc.).

Biomimetic attachment devices have already brought a lot of convenience to people’s production and life, and they will also bring great benefits to people in more fields in the future. For example, the attachment robot inspired by the remora can record video on moving objects across the air/water boundary in both the ocean and mountain streams, which may pave the way for future robots with autonomous biological detection, monitoring, and tracking capabilities in aerial–aquatic environments [22]. The new bionic mechanical arms can be used to pick apples, tomatoes, and other fruits or vegetables safely and reliably, which may be used for future agricultural harvest on a large scale in more areas and greatly improve the efficiency of agricultural production. Some advanced bionic mechanical arms can also be used in the medical field to help doctors perform difficult operations, which may reduce the burden on the doctor and improve the chances of successful surgery. The development and maturity of these devices will be conducive to the rapid development of future biomedicine. In addition, some new microsuckers can be integrated into wearable devices to monitor people’s vital signs, predict disease, help wounds recover, etc., which will let people enjoy a better quality of life.

However, there are still some difficulties and deficiencies in the research of aquatic organisms and their biomimetic attachment equipment, which are mainly:

(1) The surface microstructure of aquatic biological attachment and the regulation mechanisms of underwater attachment–detachment have not been deeply explored. Much research about attachment mechanisms is not in-depth enough. For instance, the problems of how suckermouth catfishes use their oral suction cups to achieve spontaneous breathing and attachment without interfering with each other and how abalones independently utilize multiple structures such as bristles, gully-like gastropods, and mucus to achieve autonomous control of attachment–detachment, etc. need to be solved as soon as possible.
(2) The research and development of intelligent and responsive underwater reversible attachment equipment face great challenges. Unlike on land, the underwater environment, especially the deep sea, is complex and unpredictable. When bionic attachment equipment is grasping, transporting, and monitoring in these environments, it is extremely susceptible to interference from unknown objects or stimuli. As a result, the requirements for the design and development of underwater intelligent response reversible bionic attachment equipment are significantly increased, and the research and development of related new equipment are quite challenging.
(3) The research and development process of biomimetic micro-suction cup patches with excellent characteristics such as bioapplicability, durability, and environmental protection still need to be accelerated. Although the traditional adhesive patches with high application rates have reached the standard, they still stimulate wounds and pollute the environment. At the same time, bionic micro-suction cup patches which are green, non-toxic, and of good biocompatibility can be used in wearable sensing and medical applications. However, the problems of decreased attachment and insufficient attachment time in humid environments still need to be solved as soon as possible.

Based on the above problems, the future focus and direction of biomimetic attachment research may be as follows:

(1) Continue to further explore the micro–nanostructures of aquatic biological attachment surfaces and their original attachment–detachment mechanisms, and lay a solid foundation for developing and manufacturing advanced biomimetic attachment equipment.
  Although a lot of research and exploration on the suckers of aquatic organisms have been carried out, it is not enough to only grasp the external morphological information of the structure. Combining the multi-disciplinary knowledge of physics, mechanics, biology, chemistry, etc. is necessary to study comprehensively. The mechanical properties, organizational structure, and motion control mechanisms of these aquatic organisms are characterized by many aspects [193]. Only by fully excavating the details of all aspects related to aquatic biological attachment and exploring the original attachment–detachment mechanism can we lay a good foundation for the advent of advanced biomimetic attachment equipment.
(2) Although there is a long way to go to develop multi-functional flexible underwater attachment equipment with intelligent sensing and autonomous and precise control capabilities, we must forge ahead.
  Flexible underwater robots still play an essential role in underwater transportation and monitoring. In the future, they may become the mainstay in AUV recovery, underwater rescue, all-round water, land, and air monitoring, etc. The underwater attachment equipment should eventually move toward diverse functions, intelligent response, precise control, and flexible drive sensing control integration to avoid the interference of underwater stimuli, so they can adapt to the complex underwater environment and complete their tasks more smoothly and perfectly.
(3) Continue to develop high-quality and high-performance bionic micro-suction cup patches with diverse functions, green environmental protection qualities, wide adaptability, and strong attachment capacity to better serve important fields such as wearable sensing devices and biomedical treatments.
  With the rise of non-toxic and non-polluting bionic micro-suction cup patches, they are widely used in wearable sensing devices (such as wet climbing, electronic sensing, vital sign monitoring, etc.) and biomedical treatments (such as wound dressings, rapid recovery, dermatological protection, etc.) and other fields. In addition, they may play a more critical role in the health monitoring of various organs and the controllable input of drugs in the future. However, their attachment strength on wet or rough surfaces is reduced, and the attachment time is short, which seriously limits their development. Therefore, researchers should try their best to find and test new materials and develop new suction cup patches that are more suitable for skin attachment and treatments to make up for the shortcomings of the current traditional suction cup patches.

In the future, people’s research on biomimetic attachment devices will not only rely on a more in-depth and detailed exploration of the attachment mechanism of aquatic organisms but also need the collaborative cooperation of multi-field and multi-disciplinary cutting-edge talents. In short, developing biomimetic attachment devices is full of opportunities and challenges. On the one hand, with the continuous exploration of the attachment of aquatic organisms, the mechanism of their excellent attachment performance will be more deeply revealed, and more advanced attachment mechanisms will be developed. On the other hand, the existing attachment devices still face several problems, such as narrow applicability, insufficient intelligence, etc. In the future, more intelligent attachment equipment with better attachment performance, higher reliability, wider adaptability, lower energy consumption, etc. will be developed.

## Figures and Tables

**Table 1:** Effective attachment mechanism under wet and immersion conditions [73].

Mechanisms of Adhesion	Wet Conditions (Liquid Film)	Underwater Conditions
Electrostatic forces	⊠	⊠
Van der Waals forces	⊠	⊠
Capillary forces	☑	⊠
Viscous forces	☑	⊠
Negative pressure attachment	☑	☑
Mechanical interlocking	☑	☑

(Note: ☑ indicates that it is valid; ⊠ indicates that it is essentially invalid).

## Data Availability

Not applicable.
